# Comprehensive Behavioral Analysis of Calcium/Calmodulin-Dependent Protein Kinase IV Knockout Mice

**DOI:** 10.1371/journal.pone.0009460

**Published:** 2010-03-01

**Authors:** Keizo Takao, Koichi Tanda, Kenji Nakamura, Jiro Kasahara, Kazuki Nakao, Motoya Katsuki, Kazuo Nakanishi, Nobuyuki Yamasaki, Keiko Toyama, Minami Adachi, Masahiro Umeda, Tsutomu Araki, Kohji Fukunaga, Hisatake Kondo, Hiroyuki Sakagami, Tsuyoshi Miyakawa

**Affiliations:** 1 Genetic Engineering and Functional Genomics Group, Frontier Technology Center, Kyoto University Graduate School of Medicine, Kyoto, Japan; 2 Division of Systems Medical Science, Institute for Comprehensive Medical Science, Fujita Health University, Toyoake, Japan; 3 Japan Science and Technology Agency (JST), Core Research for Evolutional Science and Technology (CREST), Kawaguchi, Japan; 4 Mouse Genome Technology Laboratory, Mitsubishi Kagaku Institute of Life Sciences, Machida, Tokyo, Japan; 5 Department of Neurobiology and Therapeutics, Graduate School of Health Bioscience, The University of Tokushima, Tokushima, Japan; 6 Department of Pharmacology, Graduate School of Pharmaceutical Sciences, Tohoku University, Sendai, Japan; 7 Laboratory for Animal Resources and Genetic Engineering, Center for Developmental Biology, RIKEN, Chuo-ku, Kobe, Japan; 8 National Institutes of Natural Sciences, Toranomon, Minato-ku, Tokyo, Japan; 9 Department of Rehabilitation, Faculty of Medical Science & Welfare, Tohoku Bunka Gakuen University, Sendai, Japan; 10 Department of Anatomy, Kitasato University School of Medicine, Sagamihara, Japan; University of Wuerzburg, Germany

## Abstract

Calcium-calmodulin dependent protein kinase IV (CaMKIV) is a protein kinase that activates the transcription factor CREB, the cyclic AMP-response element binding protein. CREB is a key transcription factor in synaptic plasticity and memory consolidation. To elucidate the behavioral effects of CaMKIV deficiency, we subjected CaMKIV knockout (CaMKIV KO) mice to a battery of behavioral tests. CaMKIV KO had no significant effects on locomotor activity, motor coordination, social interaction, pain sensitivity, prepulse inhibition, attention, or depression-like behavior. Consistent with previous reports, CaMKIV KO mice exhibited impaired retention in a fear conditioning test 28 days after training. In contrast, however, CaMKIV KO mice did not show any testing performance deficits in passive avoidance, one of the most commonly used fear memory paradigms, 28 days after training, suggesting that remote fear memory is intact. CaMKIV KO mice exhibited intact spatial reference memory learning in the Barnes circular maze, and normal spatial working memory in an eight-arm radial maze. CaMKIV KO mice also showed mildly decreased anxiety-like behavior, suggesting that CaMKIV is involved in regulating emotional behavior. These findings indicate that CaMKIV might not be essential for fear memory or spatial memory, although it is possible that the activities of other neural mechanisms or signaling pathways compensate for the CaMKIV deficiency.

## Introduction

Establishing animal models of psychiatric disorders using genetically engineered mice is essential for investigating the pathogenesis/pathophysiology and treatment of these disorders [1], [2], [3]. Previously, we reported that forebrain-specific calcineurin (also called protein phosphatase 2B) knockout (KO) mice have severe working/episodic-like memory deficits [4] and exhibit a spectrum of abnormal behaviors related to schizophrenia in humans [5]. In addition, we and others have identified a potential schizophrenia and bipolar disorder susceptibility gene, the PPP3CC gene, which encodes the calcineurin gamma subunit [6], [7], [8], [9], [10], [11]. These studies demonstrate the usefulness of subjecting genetically engineered mice to a comprehensive battery of behavioral tests to establish a mouse model of human psychiatric disorders. Therefore, we have applied this approach to various strains of mice bearing mutations of the genes encoding molecules related to either the calcineurin signaling pathways or calcineurin-related neural mechanisms [12], [13], [14], [15], [16]. Here, we focused on Ca^2+^/calmodulin dependent protein kinase IV (CaMKIV), one of the molecules that constitute calcium/calmodulin-mediated signal transduction with calcineurin [17]. Calcineurin directly dephosphorylates and inactivates CaMKIV [18]. CaMKIV phosphorylates CREB and the phosphorylation is terminated by calcineurin, possibly through direct CREB dephosphorylation or through activation of the downstream protein phosphatase 1 [19].

CaMKIV is a multifunctional, serine-threonine protein kinase that is activated in the presence of increased intracellular calcium (Ca^2+^). Compared to the ubiquitous expression of CaMKI, the tissue distribution pattern of CaMKIV is rather limited, with expression restricted primarily to discrete regions of the brain, T-lymphocytes, and post-meiotic germ cells [20], [21], [22], [23]. In the brain, CaMKIV is expressed in the hippocampus, amygdala, anterior cingulate cortex, somatosensory cortex, insular cortex and cerebellar granule cells. CaMKIV plays a role in the activity-dependent phosphorylation of CREB, which regulates the expression of genes involved in neuroplasticity [24], learning and memory [25], [26], and emotional behaviors [27], [28]. CaMKIV phosphorylates CREB at a particular residue, serine 133 (Ser133), and phosphorylation of Ser133 is required for CREB-mediated transcription [29]. Among different Ca^2+^-dependent protein kinases, CaMKIV is detected predominantly in the nuclei of neurons [30], [31], and may therefore play a unique role in the phosphorylation of CREB and in the regulation of neuronal gene expression. CaMKIV is a component of a major CREB pathway, and has therefore been hypothesized to have a significant role in synaptic plasticity and in learning and memory [32], [33], [34], [35].

To date, three independent strains of mice, including ours, with a null mutation of CaMKIV [34], [36] and transgenic mice bearing the dominant negative form of the CaMKIV gene have been created [32]. Previous studies reported the relationship between CaMKIV and fear memory [32], [33], [35], [37]. Wei et al reported that CaMKIV KO mice failed to demonstrate freezing in cued and contextual fear conditioning test [33]. Mice expressing dominant negative form of CaMKIV also exhibited deficit in cued and contextual fear conditioning test [32]. In addition, mice with CaMKIV overexpression displayed enhanced freezing response in contextual fear conditioning [35]. These studies suggest the important role of CaMKIV in fear memory. On the other hand, CaMKIV KO mice performed as well as the controls in the water maze and radial maze, suggesting intact spatial memory of CaMKIV KO mice [34]. Thus, the behavioral phenotype with regard to learning and memory of these mutant mice is not entirely consistent.

To investigate the behavioral significance of CaMKIV, we subjected CaMKIV KO mice to a battery of behavioral tests, including a neurological screen, light/dark transition, open field, elevated plus maze, social interaction, rotarod, hot plate, prepulse inhibition, Porsolt forced swim, 8-armed radial maze, Barnes circular maze, fear conditioning, and passive avoidance tests (Table 1). In previous studies, CaMKIV KO mice demonstrated impaired fear memory. Here, we used two different fear-based memory paradigms to more carefully dissect the effects of CaMKIV deficiency. Our findings indicated the usefulness of extensive behavioral testing for characterizing the involvement of specific genes and products in cognitive function.

**Table 1:** Comprehensive behavioral test battery of CaMKIV KO mice.

	Test	Age (weeks old)	Days	Result
(1st group)	GHNS	9–11	1–2	
	LD	10–12	5	Figure 1 A–F
	OF	10–12	8–10	Figure 1 M–N
	EP	11–13	11–12	Figure 1 G–L
	SI	11–12	13	Figure 2 A–E
	RR	11–13	15–16	
	HP	11–13	17	
	PPI	12–14	18–20	
	TS	12–14	23	
	PS	12–15	24–25	
	RM	15–24	45–87	Figure 4 E–H
	HC-SI	24–27	105–112	Figure 3 F
	BM	25–30	114–130	Figure 4 A–D
	HP	28–30	133	
	PA	29–32	141–148	Figure 5 E
	PS	30–32	149	
	LI	31–32	151	
(2nd group)	GHNS	12–13	1	
	HP	12–13	2	
	PS	13–15	8–16	
	FZ	21–26	68–96	Figure 5 A–D
	PA	26–31	103–132	Figure 5 F–G
	HC-SI	40–42	195–208	
	CSI	42–43	209–211	

## Results

### Generation and Characterization of CaMKIV KO Mice

**Figure 1:**
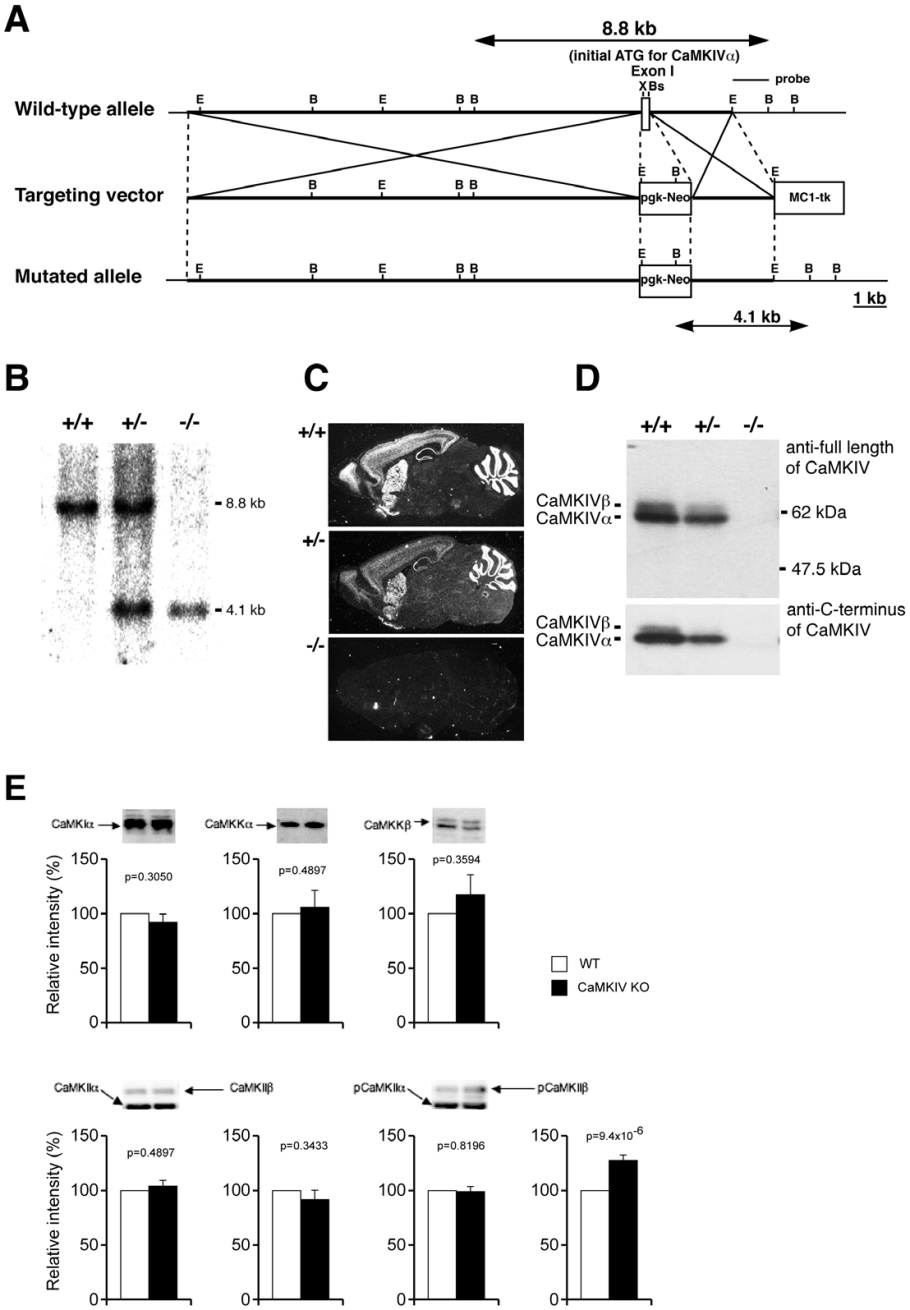
Strategy and characterization of CaMKIV gene disruption. (A) Strategy for CaMKIV disruption. B, BamHI; Bs, BstEII; E, EcoRI; X, XhoI (B) Southern blot analysis of genomic DNAs from wild-type, heterozygous, or homozygous mice with a 3′ external probe. Genomic DNAs were digested with BamHI and subjected to hybridization with radioactive 3′ probe depicted in A. (C) In situ hybridization of sagittal sections of brains from wild-type, heterozygous, or homozygous mice, showing complete attenuation of CaMKIV mRNA in CaMKIV null mouse. (D) Immunoblot analysis of brain extracts from wild-type, heterozygous, or homozygous mice, showing the absence of CaMKIV protein in CaMKIV null mice. (E) Immunoblot analysis of hippocampi extracts from wild-type and homozygous mice, showing the expression level of CaMKIα, CaMKKα, CaMKKβ, CaMKIIα, CaMKIIβ, phosphporylated CaMKIIα, and phosphporylated CaMKIIβ. Typical results of the immunoreactive bands were shown in the upper panels (wild type in the left and CaMKIV-KO in the right lanes, respectively). The quantitative results (12–16 samples for each group) were shown in the lower panels.

To disrupt CaMKIV, the exon containing an initiation codon for CaMKIVα was replaced with a neomycin resistance cassette (Fig. 1A). Southern blot analysis with a 3′ external probe confirmed the homologous recombination in CCE embryonic stem (ES) cells derived from the 129/SvJ mouse strain. Subsequently, several chimeras were generated by injecting blastocysts with the ES cells and mating C57BL/6 mice for germ line transmission. Mating of heterozygous mice generated CaMKIV-null mice at the expected Mendelian ratio (Fig. 1B). In situ hybridization analysis with a radioactive oligonucleotide probe revealed that CaMKIV mRNA was completely attenuated in brains of CaMKIV null mice and the expression level was decreased by about 50% in brains of heterozygous mice (Fig. 1C). The absence of CaMKIVα and β at the protein level in CaMKIV null mice was confirmed by immunoblot analysis with two kinds of anti-CaMKIV antibodies that were raised against different regions of the protein (Fig. 1D). We also performed immunoblot analysis using hippocampus extract with antibodies of other CaMKs to determine whether there are any compensatory changes. There were no significant differences in the expression level of other CaMKs between wild type mice and CaMKIV KO mice, suggesting that there were not any compensatory changes in the expression of these proteins (Fig. 1E). We also conducted immunoblot analysis of phospho-CaMKIIα and phospho-CaMKIIβ (Fig. 1E). There was no significant difference in the phosphorylated form of CaMKIIα between genotypes. However, the amount of the phosphorylated form of CaMKIIβ was significantly up-regulated in the CaMKIV KO mice, as compared to that in the wild type mice. There is a possibility that the increased level of phospho-CaMKIIβ may partially account for the small effect of CaMKIV deficiency on behavioral phenotype.

### Mildly Decreased Anxiety-Like Behavior and Normal Locomotor Activity in CaMKIV KO Mice

To evaluate the behavioral effects of CaMKIV deficiency, we subjected CaMKIV KO mice and their wild-type littermates to a comprehensive battery of behavioral tests (Table 1) [13]. CaMKIV KO mice appeared healthy and showed no obvious differences in physical characteristics, with the exception of a slight decrease in body weight relative to wild-type mice (WT, 27.0±0.3 g; KO, 25.4±0.3 g, genotype effect, F_1,78_ = 13.844, p = 0.0004; genotype x group interaction, F_1,76_ = 0.212, p = 0.6466). There were no significant differences between genotypes in the rotarod test, the hot plate test, acoustic startle response/prepulse inhibition, the Porsolt forced swim test, tail suspension test, or latent inhibition test (data not shown). Raw data and summary data (mean ± SEM) of the behavioral tests are shown in the mouse phenotype database (https://behav.hmro.med.kyoto-u.ac.jp/).

**Figure 2:**
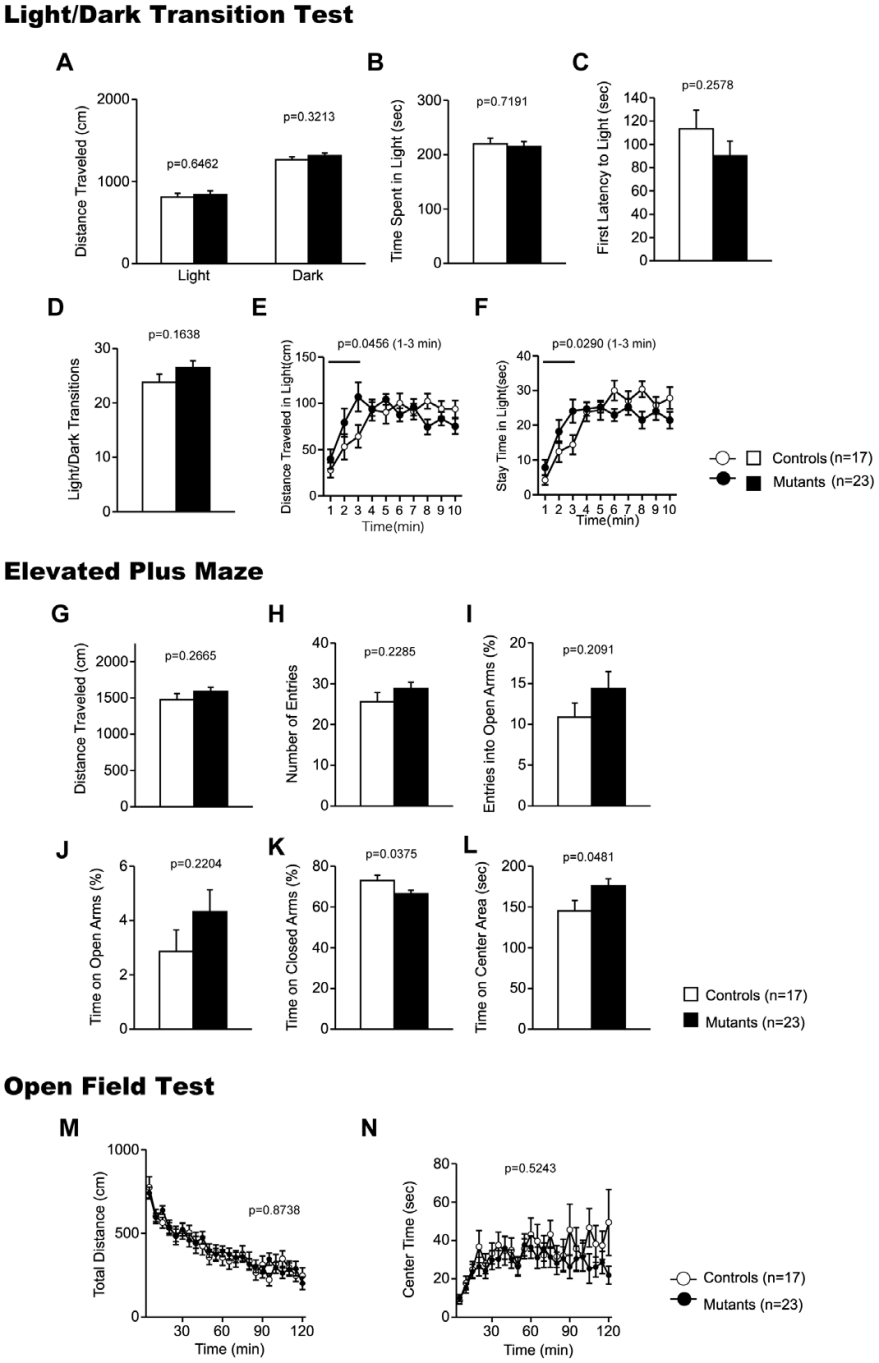
Anxiety-like behaviors in CaMKIV KO mice. (A–F) Light/dark transition test: distance traveled in the light/dark compartments (A, E), time spent in the light compartment (B, F), latency to enter the light compartment (C), and number of light/dark transitions (D) were recorded. (G–L) Elevated plus maze: distance traveled (G), number of arm entries (H), percentage of time spent on open arms (I), percentage of entries into the open arms (J), percentage of time spent in the closed arms (K), and time spent in the center area (L) were recorded. (M, N) Open field test: total distance traveled (M) and time spent in the center of the compartment (N) were recorded. The p values indicate genotype effect in two-way ANOVA (A–D, G–L) and two-way repeated measures ANOVA (E, F, M, N).

In the light/dark transition test, there were no significant differences between genotypes in distance traveled (Fig. 2A; light: F_1,38_ = 0.214, p = 0.6462, dark: F_1,38_ = 1.010, p = 0.3213), time spent in light chamber (Fig. 2B; F_1,38_ = 0.131, p = 0.7191), first latency to enter the light chamber (Fig. 2C; F_1,38_ = 1.320, p = 0.2578), or the number of transitions between the chambers (Fig. 2D; F_1,38_ = 2.016, p = 0.1638). During the first 3 min of the test, however, CaMKIV KO mice traveled significantly longer distances in the light chamber (Fig. 2D; genotype effect, F_1,38_ = 4.273, p = 0.0456) and spent more time in the light chamber (Fig. 2F; genotype effect, F_1,38_ = 5.154, p = 0.0290). These results suggest that anxiety-like behavior is mildly decreased in CaMKIV KO mice.

The results of the elevated plus maze were consistent with the notion that CaMKIV KO mice have mildly decreased anxiety-like behavior, as suggested by the results of the light/dark transition test. CaMKI KO mice spent significantly less time in the closed arms (Fig. 2K; genotype effect, F_1,38_ = 4.646, p = 0.0375) and significantly more time in the center area (Fig. 2L; genotype effect, F_1,38_ = 4.171, p = 0.0481), although there were no significant differences between genotypes in distance traveled (Fig. 2G; F_1,38_ = 1.272, p = 0.2665), number of entries into the arms (Fig. 2H; F_1,38_ = 1.498, p = 0.2285), percentage of time spent in the open arms (Fig. 2I; F_1,38_ = 1.633, p = 0.2091), and percentage of entries into the open arms (Fig. 2J; F_1,38_ = 1.553, p = 0.2204). Although, the percentage of open arm entries and the percentage of time spent in the open arms are commonly used as measures of anxiety, the time spent on the center platform of the maze and percentage of time spent in the closed arms also reflect anxiety-like behaviors in mice [38], [39]. These results also suggest mildly decreased anxiety-like behavior in CaMKIV KO mice.

Spontaneous locomotor activity was tested in the open field test. There were no significant differences between genotypes in horizontal activity (Fig. 2M; F_1,38_ = 0.017, p = 0.8971) or in time spent in the center area (Fig. 2N; F_1,38_ = 1.286, p = 0.2639). Vertical activity and stereotypic behaviors were also not significantly different between genotypes (data not shown). These results indicate that CaMKIV deficiency does not affect locomotor activity.

### Normal Social Interaction in CaMKIV KO Mice

**Figure 3:**
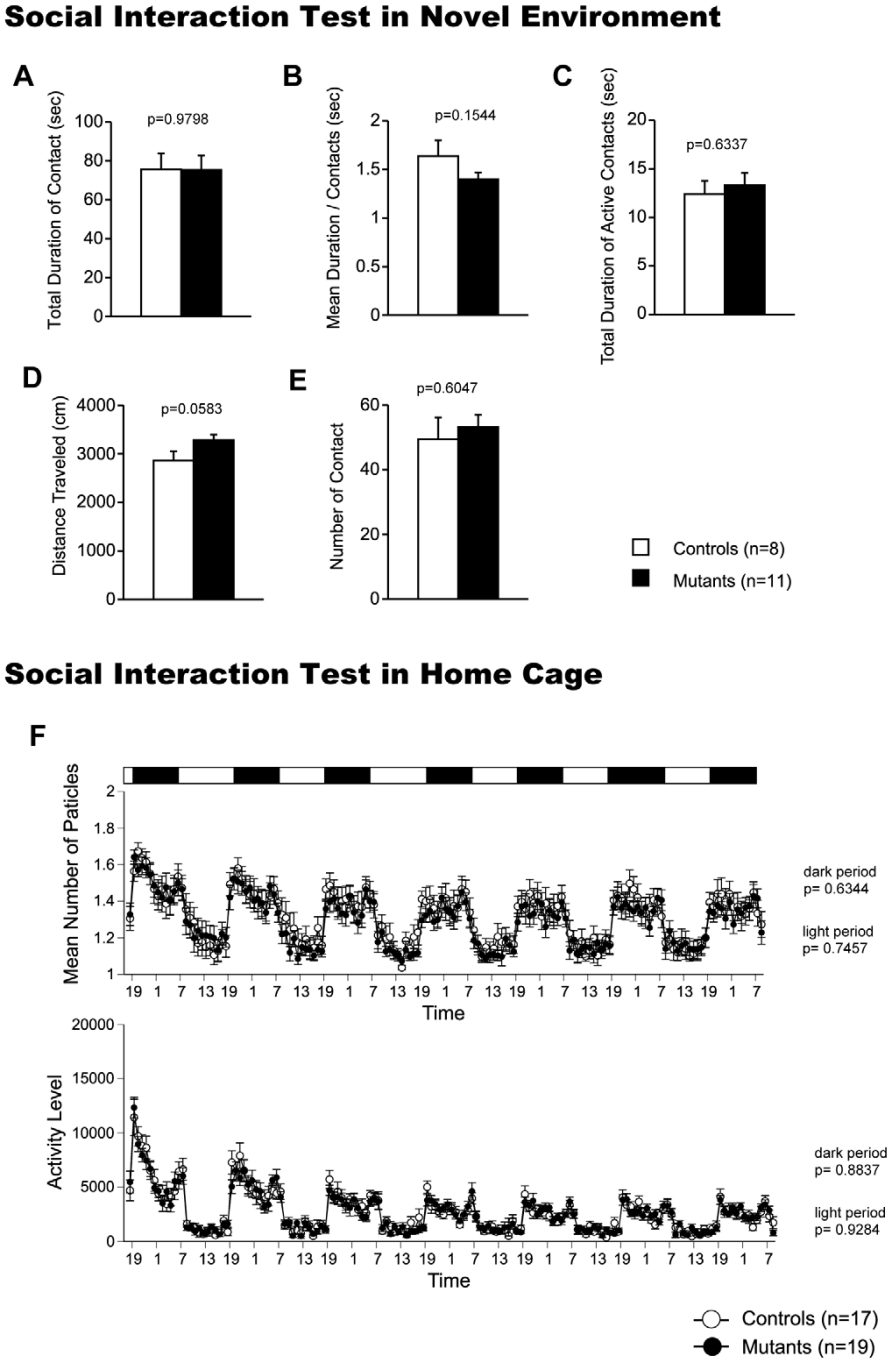
Social behaviors in CaMKIV KO mice. (A–E) Social interaction in a novel environment: total duration of contacts (A), mean duration of each contact (B), total duration of active contacts (C), total distance traveled (D), and number of contacts (E) were recorded. The p values indicate genotype effect in two-way ANOVA (A–E). (F) Social interaction in home cage: mean number of particles detected (top) and activity level (bottom) were recorded over 7 days. The p values indicate genotype effect in two-way repeated measures ANOVA.

In the social interaction test conducted in a novel environment, the total duration of contacts, mean duration per contact, total duration of active contacts, distance traveled, and mean number of contacts did not differ between genotypes (Fig. 3A–E; F_1,17_ = 0.001, p = 0.9798; F_1,17_ = 2.222, p = 0.1544; F_1,17_ = 0.236, p = 0.6337; F_1,17_ = 4.120, p = 0.0583; F_1,17_ = 0.278, p = 0.6047). We also monitored the social interaction in the home cage under familiar conditions over a 6-day period. In the social interaction test in the home cage, time spent separated is usually increased when mice are active and decreased when mice are sleeping. There were no significant differences between genotypes in the mean numbers of particles (Fig. 3F top; dark period: F_1,34_ = 0.230, p = 0.6344; light period: F_1,34_ = 0.107, p = 0.7457), indicating that CaMKIV displayed normal social interaction behavior in the home cage. The locomotor activity in the home cage did not differ between genotypes (Fig. 3F bottom; dark period: F_1,34_ = 0.22, p = 0.8837; light period: F_1,34_ = 0.008, p = 0.9284). Because we measured only activities and contacts in the social interaction tests in novel environment and in home cage, there are still the possibility that the aggressive behavior of CaMKIV KO was altered.

### Normal Spatial Learning and Memory in CaMKIV KO Mice

**Figure 4:**
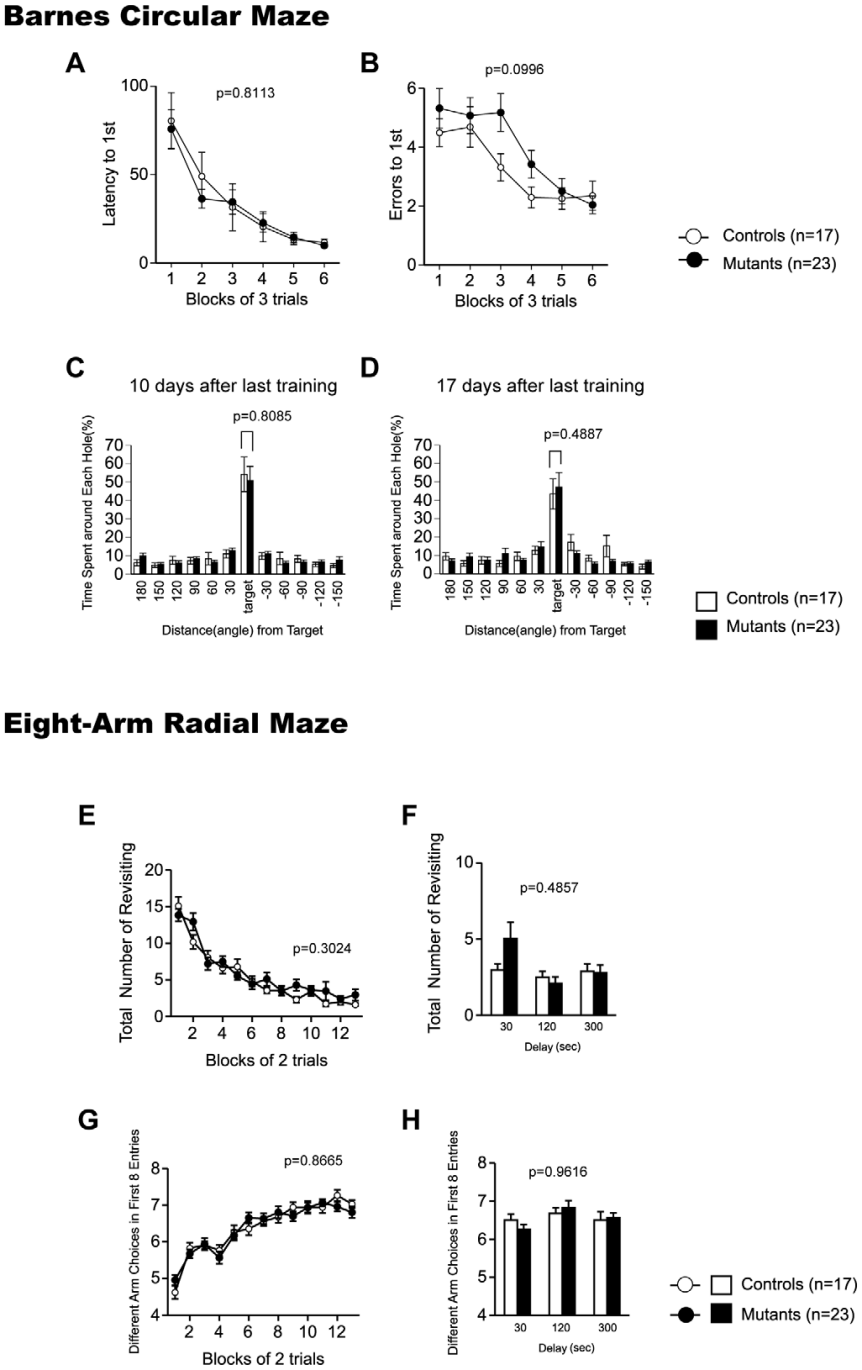
Learning and memory in CaMKIV KO mice. (A–D) Barnes circular maze: latency to reach the target hole (A), numbers of errors before reaching the target hole (B) across training were counted. Data were analyzed by two-way repeated measures ANOVA. Data are presented as means of 3 trials. The p values indicate genotype effect in two-way repeated measures ANOVA. Time spent around each hole in the probe trial conducted 24 hours (C) and 1 week (D) after last training session was recorded. The p values indicate genotype effect in two-way ANOVA. (E–H) Eight-arm radial maze: total number of arms revisited (E, F) and different arm choices among the first 8 entries (G, H) during training were recorded. During trials 27–32, a delay was applied after of the first 4 pellets were consumed (F, H). The p values indicate genotype effect in two-way repeated measures ANOVA (E, G) and two-way ANOVA (F, H).

In the Barnes circular maze, there was no effect of genotype in the latency to find the target hole (Fig. 4A; genotype effect, F_1,38_ = 0.058, p = 0.8113; genotype x trial block interaction effect, F_5,190_ = 0.465, p = 0.8020) or the number of search errors made during acquisition (Fig. 4B; genotype effect, F_1,38_ = 2.849, p = 0.0996; genotype x trial block interaction effect, F_5,190_ = 1.202, p = 0.3096), indicating normal acquisition of spatial reference memory in CaMKIV KO mice. Probe trials in which the escape box was removed were performed 1 day (1st test) and 7 days (2nd test) after the last day of training. During the probe trial, both genotypes showed a significant effect of hole location both in the 1st (Fig. 4C; location effect, WT, F_16,11_ = 15.061, p<0.0001; KO, F_22,11_ = 22.982 p<0.0001, one-way ANOVA) and 2nd test (Fig. 4D; location effect, WT, F_16,11_ = 8.533, p<0.0001; KO, F_22,11_ = 15.523, p<0.0001, one-way ANOVA), indicating that both genotypes recalled the location of the target. There were no significant differences between genotypes in the time spent around the target during the 1st and 2nd probe tests (Fig. 4C; 1st test: F_1,38_ = 0.080, p = 0.7790; Fig. 4D; 2nd test: F_1,38_ = 0.103, p = 0.7504). The results of the probe trials suggest that CaMKIV KO mice have intact consolidation/retention of spatial reference memory.

In the eight-arm radial maze test, the number of revisiting errors, in which subjects returned to the arms that had been visited previously to retrieve a food pellet, was not significantly different between genotypes during trials (Fig. 4E; without a delay: genotype effect, F_1,38_ = 1.093, p = 0.3024, genotype x trial block interaction effect, F_12,456_ = 1.491, p = 0.1236; Fig. 4F; with delays: genotype effect, F_1,38_ = 0.496, p = 0.4857). The number of different arm choices among the first 8 entries, which is considered a measure of working memory that is relatively independent of locomotor activity, and the total number of arm choices were not significantly different between genotypes (Fig. 4G; without a delay: genotype effect, F_1,38_ = 0.029, p = 0.8665; genotype x trial block interaction effect, F_12,456_ = 1.202, p = 0.2789; Fig. 4H; with delays: genotype effect, F_1,38_ = 0.002, p = 0.9616). These results demonstrate that spatial working memory is intact in CaMKIV KO mice.

### Mild Impairment of Long-Term Fear Memory in CaMKIV KO Mice

**Figure 5:**
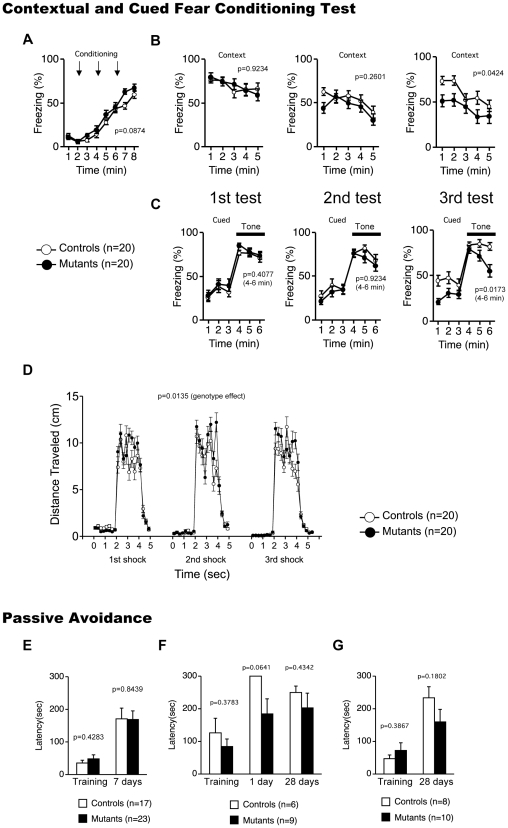
Fear memory in CaMKIV KO mice. (A–D) Contextual and cued fear conditional test: percentage of time freezing was recorded in conditioning (A), contextual (B), and cued (C) test. Distance traveled while receiving footshock was recorded (D). The p values indicate genotype effect in two-way repeated measures ANOVA. (E–G) Passive avoidance: latency to enter dark compartment was recorded. Three independent experiments were conducted. The p values indicate genotype effect in two-way ANOVA.

Mice were assessed for contextual and cued fear conditioning at 1 day, 7 days, and 28 days after exposure to a footshock paired with an auditory-conditioned stimulus. In the conditioning phase, there was no significant difference in freezing between CaMKIV KO mice and wild-type mice (Fig. 5A; genotype effect, F_1,38_ = 3.079, p = 0.0874). To examine shock sensitivity, we measured distance traveled when the footshock was delivered during training. Both CaMKIV KO mice and wild-type mice responded to footshock, but CaMKIV KO mice traveled a greater distance in response to footshock (Fig. 5D; genotype effect, F_1,38_ = 6.606, p = 0.0142). We also examined pain sensitivity using the hot plate test, and there was no significant difference in the latency to the first hind-paw response between genotypes (WT, 7.795±0.382 secs; KO, 8.320±0.456 secs, genotype effect, F_1,38_ = 0.780, p = 0.3828), suggesting normal pain sensitivity in CaMKIV KO mice. We found no significant differences between genotypes in percent time freezing in the contextual and cued test at 1 day (Fig. 5B, C left; F_1,38_ = 0.009, p = 0.9234, contextual test, F_1,38_ = 0.701, p = 0.4077, cued test) and 7 days (Fig. 5B middle; contextual test: F_1,38_ = 1.307, p = 0.2601; Fig. 5C middle; cued test: F_1,38_ = 0.773, p = 0.3850) after training. CaMKIV KO mice showed significantly less contextual freezing at 28 days after training (Fig. 5B right; genotype effect, F_1,38_ = 4.410, p = 0.0424). In the altered context, mice showed higher freezing during pre tone period (Fig. 5C). There is the possibility that few of prior experience and prior exposure to the context induced the high level of generalized freezing in the altered context during pre-tone period. There was no significant differences between genotypes in the freezing rate during pre tone period in cued test at 1 day (Fig. 5C left; F_1,38_ = 0.412, p = 0.5250) and 7 days (Fig. 5C center; F_1,38_ = 0.387, p = 0.5377) after training. Interestingly, CaMKIV KO mice displayed significantly lower freezing during the pre-tone period at 28 days after training (Fig. 5C right; F_1,38_ = 6.928, p = 0.0122), suggesting the decreased generalized fear of CaMKIV KO mice. They also displayed significantly less freezing behavior in response to the conditioned tone (Fig. 5C right; genotype effect, F_1,38_ = 6.197, p = 0.0173). These results suggest mildly impaired long-term fear memory in CaMKIV KO mice.

To further address the functional significance of CaMKIV in long-term fear memory, the mice were subjected to a passive avoidance test. There were no significant differences between genotypes in the latencies to enter the dark compartment in the training (Fig. 5E-G; F_1,38_ = 0.614, p = 0.4283; F_1,13_ = 0.832, p = 0.3783; F_1,16_ = 0.792, p = 0.3867) and the tests at 1 day (Fig. 5F; F_1,13_ = 4.092, p = 0.0641), 7 days (Fig. 5E; F_1,38_ = 0.003, p = 0.9594), and 28 days (Fig. 5F,G; F_1,13_ = 0.651, p = 0.4342; F_1,16_ = 1.964, p = 0.1802) after the training. Thus, long-term fear memory deficits were not detected in CaMKIV KO mice in the passive avoidance test. Although both the contextual and cued fear conditioning test and the passive avoidance test assess fear memory, there are some differences between the tests. In the fear conditioning test, the distance the mice travel and their freezing behavior is measured during the tests. Commonly, mice show freezing behavior when re-exposed to the conditioning chamber or to the tone delivered in the altered context. In the fear conditioning test, the indices reflect not only fear memory but also an emotional response. Because mice displayed freezing behavior in response to fear, it is possible that mice do not show high freezing even if they remember the conditioning chamber (context test) or the tone (cued test). In the passive avoidance test, on the other hand, the latency to enter the dark compartment is the only index that is obtained. Because the latency is not affected by activity or freezing of mice, memory retention is evaluated by the avoidance of the dark compartment where the footshock was delivered to the mice. CaMKIV KO mice exhibited intact memory retention in the passive avoidance task even 1 month after training.

## Discussion

In the present study, we analyzed mice with a genetic disruption of CaMKIV using a comprehensive behavioral test battery. To date, two other independent strains of CaMKIV KO mice have been created [34], [36] and their behaviors analyzed [33], [34], [40], [41]. In the previous reports, however, the mice were not sufficiently backcrossed [33], [34], [36] or KO mice were compared with non-littermate wild-type animals [40], [41]. We used mice that were backcrossed at least 10 generations into the C57/BL6N strain and analyzed using a comprehensive behavior test battery. Our results, in general, confirm and extend those of the previous studies. CaMKIV KO mice exhibited a performance deficit in a fear conditioning test. They also displayed decreased freezing in contextual and cued tests 28 days after conditioning, although there were no significant differences between genotypes 1 day and 7 days after conditioning. The lack of CaMKIV, however, did not affect performance in the passive avoidance task, another task testing fear memory, 1 day, 7 days, or 28 days after training. Furthermore, CaMKIV KO mice displayed normal spatial reference memory in the Barnes circular maze. They learned the location of the target hole as quickly as wild-type control mice. In the probe test 1 day and 7 days after the last training, both CaMKIV KO mice and wild-type control mice demonstrated a similar level of memory retention. These results suggest normal spatial reference memory of CaMKIV KO mice. We also examined spatial working memory of CaMKIV KO mice in the eight-arm radial maze. CaMKIV KO mice performed as well as controls, suggesting that spatial working memory is intact in these mice.

In a previous study, CaMKIV KO mice showed impaired memory in a contextual fear conditioning test; CaMKIV KO mice displayed less freezing behavior than control mice in both the context test and cued tests conducted 1 day and 7 days after training [33]. In our study, although we did not detect any significant differences between genotypes at 1 day or 7 days after training, CaMKIV KO mice showed less freezing behavior than control mice in both the context and cued tests at 28 days after training. Although the effect of genotype detected in the test was weak compared to that of the previous report, the effect was in the same direction. Despite some minor differences, our observation is consistent with the results reported by Wei et al that CaMKIV deficiency causes a performance deficit in the contextual and cued fear conditioning test [33]. We also tested the CaMKIV KO mice in a passive avoidance task to examine fear memory. Unexpectedly, no learning deficit was observed in the passive avoidance test, suggesting that fear memory is intact in CaMKIV KO mice, as assessed in this test.

How do we explain these seemingly discrepant results? In the fear conditioning test, memory retrieval is evaluated by freezing behavior, which is considered to reflect fear memory. In general, fearful stimuli cause freezing or fleeing in animals. Fleeing is a normal and important fear response to predators in the wild life, yet mice show freezing behavior, a passive coping behavior when strong footshocks are repeatedly applied in a fear conditioning test. If mice display attempts to escape in contextual and cued test, freezing might not be a good index for evaluating fear memory. In the passive avoidance test, fear memory can be detected in mice as an attempt to flee. Considering the differences between these tests regarding fear memory, the passive avoidance test might be more appropriate and sensitive under some conditions to detect fear memory than contextual and cued fear conditioning tests in CaMKIV KO mice.

CaMKIV deficiency resulted in mildly decreased anxiety-like behavior in the light/dark transition test and the elevated plus maze test. Shum et al also reported decreased anxiety-like behavior in CaMKIV KO mice in the elevated plus maze [40]. Those results suggest that CaMKIV deficiency causes a mild decrease in anxiety-like behavior. Decreased anxiety may attenuate freezing behavior in the contextual and cued fear conditioning tests. On the other hand, mice overexpressing CaMKIV (CaMKIV TG mice) show increased freezing behavior in a contextual fear conditioning task [37]. Wu et al analyzed only fear memory and did not assess anxiety-like behaviors, thus it is possible that anxiety-like behavior is enhanced in CaMKIV TG mice[37]. Indeed, CaMKIV TG mice showed enhanced freezing behavior after receiving electrical footshocks during the conditioning when the footshock intensity was weak [37], suggesting that CaMKIV overexpression increases shock sensitivity.

Our speculation that the extent of freezing behavior itself rather than fear memory might be modulated by CaMKIV is consistent with these reports. It is therefore possible that decreased anxiety-like behavior in CaMKIV KO mice shifted their coping behavior from a freezing response to a fleeing response and then resulted in lower non-specific freezing in the contextual test and during pre-tone and tone periods in the altered context chamber on Day 28.

We conducted two spatial learning tasks to examine spatial memory in CaMKIV KO mice. Spatial reference memory was evaluated using the Barnes circular maze and spatial working memory was tested using the eight-arm radial maze. In both tasks, CaMKIV KO mice demonstrated performances comparable to wild-type control mice, suggesting normal spatial reference and working memory in CaMKIV KO mice. Ho et al reported that CaMKIV mice showed normal spatial reference memory in the Morris water maze and normal working memory in the eight-arm radial maze [34]. Their report is consistent with our finding that spatial memory is intact in CaMKIV KO mice. On the other hand, Kang et al examined the role of CaMKIV in spatial memory and synaptic plasticity using transgenic mice expressing a dominant negative form of CaMKIV (dnCaMKIV TG mice) [32]. In contrast to the results in CaMKIV KO mice, dnCaMKIV TG mice displayed a performance deficit in the Morris water maze, suggesting impaired spatial reference memory in dnCaMKIV TG mice [32]. This discrepancy might be due to the difference between the strategies, that is, knockout of the protein and expression of the dominant negative form of the protein. In global knockout mice, compensation during development or compensation by other proteins such as CaMKI and CaMKII could occur. Such compensation could mask the role of CaMKIV in learning and memory. The strategy of expressing the dominant negative form of the protein might partially address such problems. The dominant negative form, however, could also have non-specific effects on the functions of other proteins. Although dnCaMKIV cannot bind to ATP, it does bind to Ca^2+^/calmodulin. It may trap Ca^2+^/calmodulin in the signal transduction, which may result in changes in the function of other proteins regulated by Ca^2+^/calmodulin, such as CaMKI, CaMKII, and calcineurin. These nonspecific activities of dnCaMKIV might have caused the apparent discrepancy in the results. Additionally, Kang et al used only one line of transgenic mice in their behavioral analyses [32], raising the possibility that the transgene was integrated in the genome at a locus that affects learning and memory in mice.

### Conclusion

CaMKIV KO mice display performance deficits in the contextual and cued fear conditioning tests. On the other hand, CaMKIV KO mice exhibit normal performance in the passive avoidance test and spatial learning tasks. Further, CaMKIV KO mice exhibit decreased anxiety-like behavior. Together, our results suggest that CaMKI deficiency does not cause abnormal behavior related to neuropsychiatric disorders, including schizophrenia, and also suggest that CaMKIV is dispensable for fear memory in contrast to the generally believed role of CaMKIV in learning and memory.

## Materials and Methods

### Ethics Statement

All animal care and behavioral testing procedures were approved by the Animal Research Committee, Graduate School of Medicine, Kyoto University.

### Generation of CaMKIV Mutant Mice

Genomic DNA clones containing the exon containing an initial codon of the α polypeptide of CaMKIV were isolated from a 129/SvJ mouse genomic library (Stratagene) and subcloned into the pBluescriptSK(+) vector. The targeting vector was comprised of 14.2-kb *NotI-XhoI* fragment located at 5′ of the exon, PGK-neomycin resistance gene, 4.1-kb *BstEII-EcoRI* fragment and a MC1-thymidine kinase. As a result, the exon between *XhoI* and *BstEII* sites, which contained an initial codon for CaMKIVα, was replaced with a *neo* cassette derived from pCM1*neo*-polyA (Stratagene). Linearized targeting vector was transfected into CCE ES cells [42] derived from 129/SvJ mouse strain by electroporation. Genomic DNA from ES cells selected with G418 (250 µg/ml) and gancyclovir (5 µM) were digested with *BamHI* and subjected to Southern blot analysis with the 3′ probe shown in Fig. 1A. This probe, which is a 1-kb *EcoRI/BamHI* fragment 3′ external to the targeting vector sequence, detected an 8.8-kb fragment and a 4.1-kb fragment in the case of wild-type and mutant allele, respectively. A targeted clone was injected into C57BL/6 blastcysts to generate chimeric mice. Male chimera mice were bred with C57BL/6N female mice. Mutant mice were backcrossed at least 10 generations to the C57BL/6N background.

### In Situ Hybridization Histochemistry

In situ hybridization was performed as described [43]. The oligonucleotide probe for mouse CaMKIV was 5′AGCAGGGCGAGGAGGGACAGGAGGGCACCGTGACTTTGAGCATCT3′.

### Immunoblot Analysis

Crude extracts (10 µg) of adult brains of individual genotypes were resolved and subjected to an immunoblot analysis with antibodies raised against full-length and C-terminal region (sc-1547, Santa Cruz Biotechnology, CA) of CaMKIV.

Hippcampi of mice were dissected out, put in microtube and frozen immediately in liquid nitrogen. They were homogenized in the homogenizing buffer and subjected to SDS polyacrylamide gel electrophoresis (SDS-PAGE) as described previously[44]. Proteins were transferred to PVDF membrane using Trans-Blot SD Cell (Bio-Rad) with 25 V for 1 hour. After blocking with Tris-buffered saline with Tween 20 (TTBS) containing 4.5% nonfat skim milk, membranes were incubated with the following primary antibodies: anti-CaMKIα [45] (1∶500), anti-CaMKKα [46] (1∶500), anti-CaMKKβ [46] (1∶300), anti-CaMKII [47] (1∶1000), anti-phosphorylated CaMKII [47] (1∶1000). Membranes were washed by TTBS, then incubated with the secondary antibody (anti-rabbit IgG conjugated with horse-radish peroxidase, 1∶5000, GE Healthcare). After washed by TTBS, immunoreactive bands were visualized using ECL detection kit (GE Healthcare), detected by LAS-4000 (Fuji Film), and analyzed using ImageJ (National Institute of Health). All the membranes were stained by Ponceau S (Sigma), and the major 50 kDa band which correspond to β-tubulin was used as an internal control if calibration was necessary.

### Animals and Experimental Design

CaMKIV KO mice and wild-type control littermates were obtained by breeding heterozygote mice. All behavioral tests were carried out with male mice that were at least 9 weeks old at the start of testing. Raw data of the behavioral test, the date on which each experiment was done, and the age of the mice at the time of the experiment are shown in the mouse phenotype database (https://behav.hmro.med.kyoto-u.ac.jp/). Mice were group housed (2–4 mice per cage) in a room with a 12 hr light/dark cycle (lights on at 7:00 a.m.) with access to food and water *ad libitum*. Room temperature was kept at 23±2°C. Behavioral testing was performed between 9:00 a.m. and 6:00 p.m. After the tests, all apparatus was cleaned with diluted sodium hypochlorite solution to prevent a bias due to olfactory cues. We prepared two independent groups of mice for behavioral tests. One group consisted of the equivalent number of CaMKIV KO mice and wild-type control littermates. Experiments were done in the following sequences; the first group (WT, KO; n = 17, 23): the neurological screen and wire hang (GHNS), light/dark transition (LD), open field (OF), elevated plus maze (EP), hot plate (HP), one-chamber social interaction test (SI), rotarod (RR), startle response/prepulse inhibition test (PPI), tail suspension (TS), Porsolt forced swim test (PS), latent inhibition test, eight-arm radial maze, social interaction test in home cage (HC-SI), Barnes circular maze, HP (2nd trial), passive avoidance (PA) and PS (third trial); the second group (WT, KO; n = 20, 20): GHNS, HP, PS, fear conditioning test, PA, HC-SI, Crawley's sociability and preference for social novelty test. Each behavioral test was separated from each other at least by 1 day. All behavioral testing procedures were approved by the Animal Research Committee, Graduate School of Medicine, Kyoto University.

### Light/Dark Transition Test

A light/dark transition test was conducted as previously described [48]. The apparatus used for the light/dark transition test comprised a cage (21×42×25 cm) divided into two sections of equal size by a partition with a door (Ohara & Co., Tokyo). One chamber was brightly illuminated (390 lux), whereas the other chamber was dark (2 lux). Mice were placed into the dark side and allowed to move freely between the two chambers with the door open for 10 min. The total number of transitions, latency to first enter the lit chamber, distance traveled, and time spent in each chamber were recorded by Image LD4 software (see ‘Data analysis’).

### Elevated Plus Maze Test

An elevated plus-maze test was conducted as previously described [49]. The elevated plus-maze consisted of two open arms (25×5 cm) and two enclosed arms of the same size with 15-cm high transparent walls. The arms and central square were made of white plastic plates and were elevated 55 cm above the floor. To minimize the likelihood of animals falling from the apparatus, 3-mm high Plexiglas walls surrounded the sides of the open arms. Arms of the same type were located opposite from each other. Each mouse was placed in the central square of the maze (5×5 cm), facing one of the closed arms. Mouse behavior was recorded during a 10-min test period. The number of entries into an arm, and the time spent in the open and enclosed arms were recorded. Percentage of entries into open arms, time spent in open arms (s), number of total entries, and total distance traveled (cm) were analyzed. Data acquisition and analysis were performed automatically, using Image EP software (see ‘Data analysis’).

### Open Field Test

Locomotor activity was measured using an open field test. Each mouse was placed in the corner of the open field apparatus (40×40×30 cm; Accuscan Instruments, Columbus, OH). The chamber of the test was illuminated at 100 lux. Total distance traveled (in cm), vertical activity (rearing measured by counting the number of photobeam interruptions), time spent in the center area, and beam-break counts for stereotyped behaviors were recorded. Center area was defined as 1 cm away from the walls. Data were collected for 120 min.

### Social Interaction Test in a Novel Environment

In the social interaction test, two mice of identical genotypes that were previously housed in different cages were placed in a box together (40×40×30 cm) and allowed to explore freely for 10 min [15]. Social behavior was monitored with a CCD camera connected to a Macintosh computer. Analysis was performed automatically using Image SI software (see ‘Data analysis’). The total number of contacts, total duration of active contacts, total contact duration, mean duration per contact, and total distance traveled were measured. The active contact was defined as follows. Images were captured at 1 frame per second, and distance traveled between two successive frames was calculated for each mouse. If the two mice contacted each other and the distance traveled by either mouse was longer than 2 cm, the behavior was considered as an ‘active contact’.

### Social Interaction Test in Home Cage

Social interaction monitoring in the home cage was conducted as previously described [5]. The system comprised the home cage (29×18×12 cm) and a filtered cage top, separated by a 13 cm-high metal stand containing an infrared video camera attached at the top of the stand. Two mice of the same inbred strain that had been housed separately were placed together in a home cage. Their social behavior was then monitored for 1 week. Output from the video camera was fed into a Macintosh computer. Images from each cage were captured at a rate of one frame per second. Social interaction was measured by counting the number of particles detected in each frame: two particles indicated that the mice were not in contact with each other; and one particle (i.e., the tracking software could not distinguish two separate bodies) indicated contact between the two mice. We also measured locomotor activity during these experiments by quantifying the number of pixels that changed between each pair of successive frames. Analysis was performed automatically using Image HA software (see ‘Data analysis’).

### The Barnes Circular Maze

The Barnes circular maze task was conducted on “dry land,” a white circular surface, 1.0 m in diameter, with 12 holes equally spaced around the perimeter (Ohara & Co., Tokyo). The circular open field was elevated 75 cm from the floor. A black Plexiglas escape box (17×13×7 cm), which had paper cage bedding on its bottom, was located under one of the holes. The hole above the escape box represented the target, analogous to the hidden platform in the Morris task. The location of the target was consistent for a given mouse but randomized across mice. The maze was rotated daily, with the spatial location of the target unchanged with respect to the distal visual room cues, to prevent a bias based on olfactory or the proximal cues within the maze. Three trials per day were conducted for 8 successive days. On day 9, a probe trial was conducted without the escape box, to confirm that this spatial task was acquired based on navigation by distal environment room cues. Another probe trial was conducted 1 week after the initial probe test to evaluate memory retention. Time spent around each hole was recorded by Image BM software (see ‘Data analysis’).

### Eight-Arm Radial Maze

Eight-arm radial maze test was performed using fully-automated eight-arm radial maze apparatuses [14] (Ohara & Co., Tokyo, Japan). The floor of the maze was made of white plastic, and the wall (25 cm high) consisted of transparent plastic. Each arm (9×40 cm) radiated from an octagonal central starting platform (perimeter 12×8 cm) like the spokes of a wheel. Identical food wells (1.4 cm deep and 1.4 cm in diameter) with pellet sensors were placed at the distal end of each arm. The pellets sensors were able to automatically record pellet intake by the mice. The maze was elevated 75 cm above the floor and placed in a dimly-lit room with several extra-maze cues. During the experiment, the maze was maintained in a constant orientation. One week before pretraining, animals were deprived of food until their body weight was reduced to 80% to 85% of the initial level. In the pretraining, each mouse was placed in the central starting platform and allowed to explore and consume food pellets scattered on the whole maze for a 30-min period (one session per mouse). After completion of the initial pretraining, mice received another pretraining to take a food pellet from each food well after being placed at the distal end of each arm. A trial was finished after the mouse consumed the pellet. This was repeated eight times, using eight different arms, for each mouse. After these pretraining trials, actual maze acquisition trials were performed. In the spatial working memory task of the eight-arm radial maze, all eight arms were baited with food pellets. Mice were placed on the central platform and allowed to obtain all eight pellets within 25 min. A trial was terminated immediately after all eight pellets were consumed or 25 min had elapsed. An ‘arm visit’ was defined as travelling more than 5 cm from the central platform. The mice were confined at the center platform for 5 s after each arm choice. The animals went through one trial per day. For each trial, arm choice, latency to obtain all pellets, distance traveled, number of different arms chosen within the first eight choices, the number of arm revisited, and omission errors were automatically recorded. Data acquisition, control of guillotine doors, and data analysis were performed by Image RM software (see ‘Data analysis’).

### Contextual and Cued Fear Conditioning Test

Each mouse was placed in a test chamber (26×34×29 cm) inside a sound-attenuated chamber and allowed to explore freely for 2 min. A 60 dB white noise, which served as the conditioned stimulus (CS), was presented for 30 secs, followed by a mild (2 secs, 0.5 mA) footshock, which served as the unconditioned stimulus (US). Two more CS-US pairings were presented with a 2-min inter-stimulus interval. Context testing was conducted 24 h, 7 days and 28 days after conditioning in the same chamber. Cued testing with altered context was conducted after conditioning using a triangular box (35×35×40 cm) made of white opaque Plexiglas, which was located in a different room. The chamber of the test was illuminated at 100 lux. Data acquisition, control of stimuli (i.e. tones and shocks), and data analysis were performed automatically, using Image FZ software (see ‘Data analysis’). Images were captured at 1 frame per second. For each pair of successive frames, the amount of area (pixels) by which the mouse moved was measured. When this area was below a certain threshold (i.e., 20 pixels), the behavior was judged as ‘freezing’. When the amount of area equaled or exceeded the threshold, the behavior was considered as ‘non-freezing’. The optimal threshold (amount of pixels) to judge freezing was determined by adjusting it to the amount of freezing measured by human observation. ‘Freezing’ that lasted less than the defined time threshold (i.e., 2 secs) was not included in the analysis. The parameters were constant for all mice assessed.

### Passive Avoidance Test

The apparatus was a trapezoidal box, consisting of one dark and one bright chamber connected by a guillotine door (Ohara & Co., Tokyo). Each mouse was first placed into the lighted chamber and the guillotine door was opened. After mouse entered the dark chamber, a 2-sec footshock at 0.3 mA was delivered to mouse. Mice that did not enter the dark chamber within 300 sec were excluded from analysis. One day, 1 week, and 1 month later, animals were tested for retention by placing each animal into the lighted chamber and the latency of the mouse entering the dark chamber was recorded.

### Neurological Screen

Neurological screen was performed as previously described [14]. The righting, whisker touch, and ear twitch reflexes were evaluated. A number of physical features, including the presence of whiskers or bald hair patches, were also recorded.

### Hot Plate Test

The hot plate test was used to evaluate sensitivity to a painful stimulus. Mice were placed on a 55.0 (±0.3)°C hot plate (Columbus Instruments), and latency to the first hind-paw response was recorded. The hind-paw response was defined as either a foot shake or a paw lick.

### Rotarod Test

Motor coordination and balance were tested with the rotarod test. The rotarod test, using an accelerating rotarod (UGO Basile Accelerating Rotarod), was performed by placing mice on rotating drums (3 cm diameter) and measuring the time each animal was able to maintain its balance on the rod. The speed of the rotarod accelerated from 4 to 40 rpm over a 5-min period.

### Tail Suspension Test

The tail suspension test was performed for a 10-min test session. Mice were suspended 30cm above the floor in a visually isolated area by adhesive tape placed ∼1cm from the tip of the tail, and their behavior was recorded over a 10-min test period. Data acquisition and analysis were performed automatically, using Image TS software (see Section ‘Data analysis’).

### Porsolt Forced Swim Test

The apparatus consisted of four plastic cylinders (20 cm height ×10 cm diameter). The cylinders were filled with water (23°C) up to a height of 7.5 cm. Mice were placed into the cylinders, and their behavior recorded over a 10-min test period. Data acquisition and analysis were performed automatically, using Image PS software (see ‘Data Analysis’). Distance traveled was measured by Image OF software (see ‘Data Analysis’) using stored image files.

### Latent Inhibition Test

On the first day, each mouse was placed in a conditioning chamber (O'hara & Co., Tokyo). The mice were divided into two groups: pre-exposed (P) group and non pre-exposed (NP) group. The P group received 40 white noise tones (55 dB, 5 s duration, 25 s interstimulus interval), whereas the NP group received no stimulus during an equivalent period. Immediately after the tone pre-exposure or the exposure to the chamber, tone-shock pairs consisting of a 5-s tone coterminating with a 2-s foot shock at 0.25 mA were delivered to both groups with a 25-s interstimulus interval. Afterward, mice remained in the chamber for 25 s before being returned to the home cage. On day 2, the mice were placed back in the conditioning chamber for 5 min for the measurement of freezing to the context. On the same day, the mice were put in a triangular box (35 cm ×35 cm ×40 cm) made of white opaque Plexiglas, which was located in a different room, and after 180 s, a 180-s tone was delivered to measure cued freezing.

### Crawley's Sociability and Social Novelty Preference Test

Crawley's sociability and social novelty preference test is well-designed method to investigate the complex genetics of social behaviors. The social testing apparatus consisted of a rectangular, three-chambered box and a lid with an infrared video camera (Ohara & Co., Tokyo). Each chamber was 20 cm ×40 cm ×22 cm and the dividing walls were made from clear Plexiglas, with small square openings (5 cm ×3 cm) allowing access into each chamber. An unfamiliar C57BL/6J male (stranger 1), that had had no prior contact with the subject mice, was placed in one of the side chambers. The location of stranger 1 in the left vs. right side chamber was systematically alternated between trials. The stranger mouse was enclosed in a small, round wire cage, which allowed nose contact between the bars, but prevented fighting. The cage was 11 cm in height, with a bottom diameter of 9 cm, vertical bars 0.5 cm, and horizontal bars spaced 1 cm apart. The subject mouse was first placed in the middle chamber and allowed to explore the entire social test box for a 10-min session. The amount of time spent in each chamber was measured with the aid of a camera fitted on top of the box. Each mouse was tested in a 10-min session to quantify social preference for the first stranger. After the first 10-min session, a second unfamiliar mouse was placed in the chamber that had been empty during the first 10-min session. This second stranger was also enclosed in an identical small wire cage. The test mouse thus had a choice between the first, already-investigated unfamiliar mouse (stranger 1), and the novel unfamiliar mouse (stranger 2). The amount of time spent in each chamber during the second 10-min was measured as described above. Data acquisition and analysis were performed automatically, using Image J based original program (see ‘Data Analysis’) software.

### Data Analysis

Behavioral data were obtained automatically by applications based on the public domain NIH Image program and Image J program and modified for each test by Tsuyoshi Miyakawa (available through Ohara & Co.). Statistical analysis was conducted using StatView (SAS Institute, Cary, NC). Data were analyzed by two-tailed t-test, two-way ANOVA, or two-way repeated measures ANOVA. Values in graphs are expressed as mean ± SEM.
